# The Role of Health Care Communication in Treatment Outcomes

**DOI:** 10.1146/annurev-linguistics-030521-054400

**Published:** 2022-10-07

**Authors:** Tanya Stivers, Alexandra Tate

**Affiliations:** 1 Department of Sociology, University of California, Los Angeles, California, USA; 2 Department of Medicine, University of Chicago, Chicago, Illinois, USA

**Keywords:** physician–patient, conversation analysis, negotiation, decision making, social interaction

## Abstract

The physician–patient relationship has evolved significantly in the past century. Physician authority has been reduced while patients have been empowered. This review focuses on face-to-face clinical care and argues that current physician–patient relations range from partnerships between social actors who each play critical roles in negotiating care to a more adversarial duel in which both participants advocate for goals that are not necessarily shared. While the former is the hope of increased patient involvement, the latter is increasingly common. Through our discussion of existing studies, we document that while high levels of patient participation are beneficial to treatment outcomes, this engagement also has a dark side that threatens treatment outcomes. We discuss some communication resources patients use that affect treatment outcomes, exemplify how patient engagement affects physician communication, and discuss some strategies that current research finds effective for communicating about treatment with today’s engaged patients.

## THE ROLE OF PHYSICIAN–PATIENT COMMUNICATION IN TREATMENT OUTCOMES

The physician–patient relationship has evolved in the past century. Over this time, we have seen significant changes in medical authority, and the role of patients has also changed. Patient empowerment, consumerist movements, and policy changes have created a context of more patient-centered health care (Timmermans 2020). This review focuses on research that looks directly at face-to-face clinical care with an emphasis on treatment outcomes, mostly in the United States. We argue that the present state of physician–patient relations manifests in communication that ranges from a partnership between social actors who each play critical roles in negotiating care to a more adversarial duel in which both participants advocate for goals that are not necessarily shared. While the former is the hope of increased patient involvement, the latter is increasingly common. Through our discussion of existing studies, we document that treating engaged patients has key benefits due to their levels of engagement but that this level of engagement also has a dark side that threatens the continuation of positive treatment outcomes. We discuss what health communication practices look like in today’s system and the tools that physicians have to reap the benefits of patient participation while also managing potentially negative consequences of some participation strategies.

## THE CHANGING PATIENT ROLE

In medical sociology, physicians’ professional authority has been understood as rooted in their possession of knowledge, skills, and access to drugs and techniques that patients do not possess (Parsons 1937). Specifically, physicians have the knowledge to diagnose and understand what the diagnostic implications are for treatment, and they possess the legal and cultural authority to prescribe medicines and order diagnostic tests (Starr 1982, Timmermans & Oh 2010). Thus, physicians are conceptualized as having both epistemic and deontic authority over patients’ treatment. Epistemics refers to “the distribution of rights and responsibilities regarding what participants can accountably know, how they know it, whether they have rights to describe it” (Heritage & Raymond 2005, p. 18). Clinicians’ epistemic authority is rooted in knowledge of the human body, disease processes, and treatments (Whooley 2013). It is their professional authority to recommend changes in patients’ behavior that allows for deontic authority: the “right to determine others’ future actions” (Stevanovic & Perakyla 2012, p. 297). In the clinical context, physicians exercise authority on deontic grounds when they recommend medications, tests, or changes in lifestyle.

Both dimensions of physician authority were enhanced by state protections of medicine and institutional safeguards of the practice of medicine (Abbott 1988, Light 2010, Menchik & Jin 2014, Starr 1982, Timmermans & Oh 2010). In a context of high medical authority and patients with little access to medical knowledge, a paternalistic communication style prevailed in clinical practice across the 1950s and 1960s: Physicians asserted their views and recommendations with little insight into the bases for their recommendations while patients generally offered little to no resistance of diagnoses or treatment recommendations (Byrne & Long 1976).

However, this model was heavily criticized in the 1970s and 1980s. As decreasing health costs and broader access to health care became priorities, physicians came under fire (Light 2010). Moreover, scholars began to hold paternalistic communication responsible for high rates of patient nonadherence to treatment regimens (Bruner et al. 1976, Byrne & Long 1976, Roter 1977).

In addition to pressures to reduce physicians’ professional authority came pressures to increase the patient’s role in treatment decisions (Halpern 2004). After all, patients were already becoming recognized as playing a more active role in their care because ultimately, they decide whether to take pills, come in for X-rays, and measure their blood glucose levels. If there was more patient buy-in, perhaps patients would be more willing to follow advice (Korsch & Negrete 1972). Taken together, the pressures against physician authority and for patient involvement created a push for deontic authority to be shared between physicians and patients regardless of disparities in epistemic authority.

This policy turn towardpatient empowerment (Comm. Hosp. Care & Inst. Patient Fam.-Cent. Care 2012, Emanuel & Pearson 2012) encompasses many forms of health communication, but with regard to treatment, it usually involves physicians providing patients with all pertinent information about their conditions, engaging them in decision making, addressing the fall range of biopsychosocial manifestations of illness and disease, and building alliances based on shared power and responsibility (Mead & Bower 2000). Conversely, patients should offer their preferences, values, beliefs, and burden tolerances (Charles et al. 1999).

While physicians are required to mobilize their expertise in shared decision making through communicating treatment preferences, the model nonetheless represents an erosion of physician authority insofar as it runs counter to the model of paternalistic decision making through promoting patient autonomy and choice (Brown et al. 2004). In this care model, the decision-making process is conceptualized as collaborative: Physician and patient are involved in treatment decisions through two-way exchanges of information, preferences, and investment in the treatment decision (Legare & Thompson-Leduc 2014). Known as the gold standard in medical decision making, this type of decision making was among many of the key reforms to the US health care system when the Patient Protection and Affordable Care Act was signed into law in 2010. Many states have also endorsed collaborative decision-making practices and have put forth policies intended to encourage patient involvement in medical decisions (Frosch et al. 2012).

Augmenting this policy push for patient involvement in health care was the rise of consumerism encouraging greater individual autonomy, even in health care (Lupton 1997). While these pressures encourage patient involvement, the Internet added a final critical component by giving patients access to all sorts of medical information that they are bringing to bear on their care decisions (Akerkar & Bichile 2004, Boyer & Lutfey 2010, Stevenson et al. 2021, Wald et al. 2007).

While most of the time, physicians maintain clear epistemic primacy over diseases, the human body, and treatment, today’s patients have substantial epistemic access to the underlying information, which allows them to gain epistemic authority. That combined with the policy push for a reduction in the asymmetry of deontic authority has made visible a shift from a more authoritarian model of care to a provider-client model, one that has become more egalitarian over time (Timmermans 2020).

Still, this shift should not be overstated: An asymmetry in the clinician-patient relationship remains for reasons that are both patient- and physician-driven. One account for the persistent asymmetry is that physicians have not only lost but also gained authority due to innovations and developments in the medical knowledge base (Timmermans & Mauck 2005). As the scope of medical science has expanded to embrace effective and reliable treatment, so too has physician authority (Cutler 2004). Patient behavior also helps to maintain physicians’ medical authority. For instance, some patients remain wary about participating in medical decisions (Levinson et al. 2005). Moreover, whether they are actually reticent or not, most patients still do not contest physicians’ diagnostic claims or treatment recommendations (Stivers et al. 2018). Despite wide public availability of medical information, the physician–patient knowledge gap persists.

Scholars of clinical communication have identified a wide array of indicators of this more egalitarian model in effect in the medical encounter. While some of these indicators are positive and embody the kind of patient involvement in treatment that was envisioned by advocates of patient-centered care or shared decision making, others are better described as unintended consequences of this shift in care. These communication practices range from more proactive ways that patients present their problems or answer clinicians’ questions, to patients asking questions, advocating for specific diagnoses and treatments, and resisting or even outright rejecting physicians’ views and treatments.

In what follows, we review some of the ways that patient involvement shapes treatment outcomes, sometimes in ways that promote patient health, and other times in ways that are potentially detrimental to patient health. We then turn to a discussion of how increased patient participation has shaped physician communication. Third, we identify some of the communication practices that can help preempt or mitigate situations where patients are advocating for treatment that may be unwarranted or ill advised. Finally, we discuss what else could be done through communication tools and structures of health care to further improve patient care. Our review focuses on studies that rely on video and audio recordings of medical visits.

## HOW PATIENT INVOLVEMENT SHAPES TREATMENT OUTCOMES

The myriad ways that patients influence treatment is a sign of the shift in health care to more proactive and involved patients. Understanding the sources of patient influence is important to maintaining high-quality health care in addition to learning more about the evolving provider–patient relationship. We begin by considering the role of patients advocating for medication (Kravitz et al. 2005, Wang 2020). We then discuss more subtle ways of influencing clinicians during patients’ descriptions of their problems, before receiving a diagnosis, and after diagnosis. These more subtle strategies are both more prevalent and more likely to be missed by scholars and clinicians alike despite their effectiveness.

### Advocating for Treatment

Direct requests for treatment constitute clear evidence that patients are willing and able to challenge medical authority. After all, a hallmark of medical authority is the ability to decide a course of treatment and provide that recommendation to the patient. While no recording-based study of outright requests exists from medical consultations during the 1950s and 1960s, based on the information we have from data such as those of Byrne & Long (1976) from the United Kingdom and Korsch & Negrete (1972) in the United States, we can surmise that such requests were exceedingly rare in the more paternalistic communication context of that period.

Today’s patients do make requests—requests for referrals, tests, antibiotics, antidepressants, and antianxiety medications, to name a few. However, outright requests for medicines remain un-common in primary care. For instance, out of 138 consultations for moderate to severe pain, only 1 patient requested medication (Timmermans et al. 2022). In adult consultations for upper respiratory infections, out of 68 visits, 1 patient requested antibiotics (Stivers & Timmermans 2021).

Other forms of overt lobbying such as statements of need or claims about past effectiveness of medications are more frequent. In upper respiratory cases among adults, such lobbying occurred in 13% of cases (9/68) (Stivers & Timmermans 2021). Among visits for children, the rate was 9% of cases (32/360) (Stivers 2002). In psychiatric visits, overt solicitations appear more common, occurring in as many as 42% of visits in one study (15/36) (Bolden et al. 2019).

Patients who make requests or otherwise overtly lobby for a medication consistently influence prescribing outcomes. For instance, in a randomized controlled trial, Kravitz et al. (2005) used standardized patients and found that the ways in which patients with mental health symptoms requested psychotropic drugs (no request for medication versus general request for medication versus specific brand request) shaped how physicians treated their symptoms. Patients who made a general or a brand-specific request for medicine received a prescription and/or referral to a specialist significantly more often than patients who identified their symptoms without a request for medication. Relatedly, when parents overtly lobby for antibiotics in contexts where such medications are not clinically indicated, they at times change the course of treatment (Stivers 2002, Stivers & Timmermans 2021).

There is little doubt that patients’ overt lobbying for medication can influence a physician’s prescribing trajectory, but these behaviors remain relatively infrequent. Thus, while the sheer presence of such behaviors represents a shift in the nature of the physician–patient relationship from paternalistic to relatively egalitarian, their scarcity speaks to physicians’ retention of medical authority. In this context, what is striking is how patients more subtly influence clinicians in ways that are less overtly threatening of medical authority while still effective in shaping treatment outcomes.

Patients perform a wide range of actions that steer physicians toward (or away from) particular diagnostic or treatment outcomes. Patients’ communication practices range from “priming” the physician to prescribe a particular treatment through language in their problem presentations to resisting recommended treatments, and many behaviors in between (Stivers & Timmermans 2021). These proactive behaviors are critical in two respects: First, they represent a perhaps unforeseen form of patient participation where patients advocate for diagnoses and treatments that may not be in line with clinical evidence. Second, they shed light on the current state of the physician–patient relationship, suggesting that medical authority is being undermined through these actions but being upheld through the design of the actions and their limited frequency.

### The Problem Presentation

One of the earliest points at which patients can shape the course of the medical visit is when they characterize their problems. This is particularly true for encounters regarding new conditions. However, even in chronic care visits, patients can describe their current state in alternative ways. How they do so matters for treatment outcomes. For instance, patients who present their symptoms as the natural result of social stress are less likely to receive antidepression medication than those who focus on the symptoms (Karasz et al. 2012).

Relatedly, in primary care consultations where patients identify mental health issues, when patients directly mention that these symptoms are tied to lifeworld disruptions such as their ability to care for their house or work, they are more likely to be treated than when they do not make these connections explicitly, even if the symptoms are otherwise presented at comparable levels of severity (Tate 2019). The specific link to a lifeworld disruption is also more likely to lead to prescription medication.

In the arena of upper respiratory tract infections, when patients or caregivers present the problem by naming a bacterial infection as a candidate explanation for the symptoms (i.e., a candidate diagnosis), physicians are more likely to understand patients as pressuring for antibiotics (Stivers 2002) and to subsequently prescribe (Stivers et al. 2003). For instance, a patient who uses language like “I have a sore throat” or “I cough a lot, especially at night” is less likely to be perceived as expecting an antibiotic than a patient who uses language like “I’m worried it’s strep throat” or “I am prone to pneumonia.” By invoking a diagnosis, even one like pneumonia that can be both viral and bacterial, physicians tend to perceive parents and patients as steering them toward an antibiotic via a bacterial diagnosis.

### Before the Diagnosis

Patients also influence treatment outcomes through communication strategies that are not restricted to the problem presentation. For instance, by invoking extenuating personal circumstances (e.g., “He has his big 5-year-old birthday party”; “I have a big case at work”; “We’re leaving for vacation on Monday”) that seemingly have nothing to do with accurately diagnosing a patient’s upper respiratory symptoms, patients invite physicians to see a link. This is typically the need for a quick fix so that patients can enjoy the birthday party, fulfill their obligations, or comfortably take a flight (Stivers & Timmermans 2021).

Physicians’ questions about patients’ symptoms and prior efforts to self-treat also provide opportunities for patients to subtly advocate for diagnoses or treatments. For instance, the polarity of the questions doctors ask (toward yes or no) can reveal their stances toward a patient’s health issue (Boyd & Heritage 2006). Through “optimization” (i.e., polarizing questions toward confirming routine, no-problem answers), physicians project a no-problem diagnosis. In the context of an acute concern such as an upper respiratory tract infection, physicians typically “optimize” the design of unusual, more serious, and unmentioned problems by putting them in the negative (e.g., “So no blood in the stool?”; “You’re not coughing up blood, right?”). By contrast, they design questions about typical or mentioned symptoms in an affirmative, problem-attentive way, such as “You’ve been coughing?” or “Does your throat hurt?”

A physician’s series of questions can reveal a diagnostic trajectory. For instance, “So no fever?” followed by “And this has been going on just three days now?” and then “And the coughing is mostly at night?” all point toward a routine viral cold diagnosis because of the absence of fever, the short duration, and a cough that is brought on by postnasal drip. In these questioning contexts, patients sometimes exploit the opportunity to nudge physicians away from a no-problem/no-treatment trajectory (Stivers & Timmermans 2021). For instance, in responding to a physician’s question about whether a boy is coughing “a lot,” the parent disconfirms but does so not by saying “No” but by working to send the physician down a new diagnostic trajectory with “Not a lot but it sounds deep” (Stivers 2007). This type of nudging in the context of upper respiratory tract infections is associated with inappropriate antibiotic prescribing (Stivers 2021).

Along similar lines, a doctor’s questions during a search for pain can give a patient insight into where the diagnosis is headed, allowing a patient to shape diagnostic outcomes (McArthur 2019). For instance, when doctors use a turn-initial question and physical touch to attempt to locate the source of the patient’s pain, they may not get it right. Patients influence doctors in their searches by directing them to alternate locations and embodying pain displays when doctors get closer.

Relatedly, in visits where clinicians explain the results of exome testing to families, parents can shape whether a genetic variant of uncertain clinical significance is ultimately regarded as uncertain or rather as having likely (or not likely) caused the child’s condition. For instance, when a physician presents an uncertain interpretation and the parent then introduces information about parents’ symptoms that was not previously discussed but is supportive of a causal analysis, this can lead the clinician toward a more causal interpretation for the child (Stivers & Timmermans 2016).

Another form of patient steering that influences prescribing is patients’ references to “medical misdeeds” (Bergen & Stivers 2013). In these cases, patients introduce their own failures of treatment adherence or their taking of a spouse’s prescribed medications without physician permission. While patients do not orient to such references as lobbying for medications per se, these disclosures nonetheless function as pressure because they convey to physicians that patients will follow the treatment they want if physicians’ treatment recommendations are out of line with their own preferences. Thus, a patient who has used a spouse’s prescription pain medicine and tells the physician she has done so to help her sleep is given a prescription sleep aid in her visit.

Patients steer physicians toward and away from diagnoses and treatments in still other ways too. Gill & Maynard (2006) discussed how patients introduce lay explanations for their illnesses (e.g., a period causing depression, fatigue being due to overwork, back pain being the result of driving a different car). In these instances, physicians most commonly confirm patients’ explanations, albeit cautiously.

In this section, we have reviewed some of the many ways patients shape treatment trajectories through their communication practices before physicians provide diagnoses. Unlike a problem solicitation, which provides a platform for patients to characterize their problems as they wish (e.g., through a narrative, a list of symptoms, or diagnostic theories), during the verbal and physical examinations, physicians typically ask a series of tightly designed questions. Still, patients take these opportunities to weave in explanations, additional points, and/or assessments and can shape treatment outcomes.

### After the Diagnosis

Once a clinician provides a diagnosis to a patient, any patient work to modify the clinician’s recommended treatment trajectory will be heard as relatively combative. This sets these communication practices apart from most of those we have reviewed thus far. In contrast to the subtle steering of physicians through problem presentations, answers to questions, or mentions of extenuating circumstances or alternative diagnostic theories, after they have received diagnoses or treatment recommendations, patients’ primary communication resource for altering treatment outcomes is resistance.

Resistance generally entails disagreeing with or questioning physicians’ diagnoses or treatments. However, there are differences between diagnosis resistance and treatment resistance. Specifically, after a physician’s diagnosis, no response from the patient is normatively required. Thus, silences, continuers like “Uh huh” or “Mm hm,” and acknowledgments such as “Got it” are not understood as resistant actions. Instead, only active questioning, challenging, or disagreeing constitutes diagnosis resistance. Questioning a physician’s diagnosis may involve a direct but small query such as “Really?” or “It is?” but it can also involve challenges that take physicians back to their examinations, such as “Did you check her throat?” Viral diagnosis resistance in the context of upper respiratory tract infections is associated with physicians perceiving parents as expecting antibiotics and thus constitutes pressure for antibiotics (Stivers et al. 2003).

Patients’ ability to shape encounter outcomes is particularly robust in the domain of treatment recommendations, where there is a shared orientation to patients having a normative obligation to endorse such recommendations (Koenig 2011, Lindström & Weatherall 2015, Stivers 2005b). Physicians and patients alike see the physician’s provision of a treatment recommendation as proffering a plan for patient acceptance or rejection. Evidence comes first from the consistency with which patients provide acceptance. Absent resistance, when physicians announce diagnoses, patients rarely do more than acknowledge them except when resisting. However, when it comes to treatment plans, it is common for patients to accept with “Okay” or “Alright” (Stivers 2005b).

A second prong of evidence for the relevance of patient acceptance in the discourse of treatment recommendations is the fact that when patients do not offer an “Okay” or “Alright,” physicians pursue patient acceptance. For instance, once physicians have recommended for or against a treatment, if the patient does not explicitly and vocally accept it, physicians consistently go on to offer accounts for why their recommendation is good, or they directly solicit acceptance (e.g., by asking, “Does that sound okay?”). Some physicians even pursue acceptance through changes in the treatment plan (Stivers 2005b).

Taken together, whereas silences and continuers are not routinely understood as resistance in the context of diagnoses, they are heard as resistant after the delivery of treatment recommendations. Thus, treatment resistance can be either passive or active, with passive resistance commonly preceding active resistance (Stivers 2005b). Moreover, while active resistance consists of questioning, challenging, or even outright rejection of physicians’ recommendations, questions such as “So there’s no medicine?” are more common than rejections or refusals of recommendations. Rates of active patient resistance range from 10% to 22% in primary care (Mangione-Smith et al. 2006, Stivers et al. 2018) to as high as 40% in pediatric neurology (Stivers & Timmermans 2020). As with viral diagnosis resistance, nonantibiotic treatment resistance in the context of upper respiratory tract infections is also associated with physicians’ perceptions of parents as expecting antibiotics (Mangione-Smith et al. 2006).

All of this is important because treatment resistance has been shown to affect a range of different prescribing outcomes not only in the United States but also in the United Kingdom, Brazil, China, and Japan (Barton et al. 2016, Kushida & Yamakawa 2015, Nielsen 2019, Ostermann 2021, Pilnick & Coleman 2003, Stivers 2021, Stivers et al. 2003, Wang 2020). For instance, in the realm of antibiotic prescribing for children, when parents resist a nonantibiotic treatment recommendation, both US and Chinese physicians are more likely to prescribe antibiotics inappropriately (Mangione-Smith et al. 2006, Wang 2020). In pediatric neurology, physicians who encounter resistance to their recommendations for medication changes will sometimes yield to parent resistance—sometimes until the next visit, other times offering an alternative treatment to their original recommendation (Stivers & Timmermans 2020). In Japanese psychiatry visits, patient resistance can also shape treatment outcomes in ways that are more attuned to patient preferences (Kushida & Yamakawa 2020).

Relatively little is known about the extent to which individuals from particular socioeconomic or cultural backgrounds engage in resistance or other forms of pressure. However, African American parents in one study never resisted a physician recommendation against antibiotics, whereas parents of all other racial and ethnic groups did so (Mangione-Smith et al. 2006).

Overt lobbying, problem presentation design, prediagnosis statements, and patient resistance (of the diagnosis or the treatment recommendation) all represent key communication practices through which patients shape treatment outcomes. The types of outcomes associated with these behaviors clearly constitute patient participation in the medical visit. However, such behaviors are not likely the sort of patient engagement originally envisioned by proponents of patient-centered care policy. This is not so much because patients are shaping treatment outcomes but because the outcomes that they are shaping are not necessarily in line with best clinical practice. For instance, when these sorts of communication practices lead to clinicians prescribing antibiotics or opioids where they are not clinically indicated or not prescribing chronic care medication where they are indicated, there is a conflict between patient engagement and standards of care.

## CONSEQUENCES OF PATIENT ENGAGEMENT FOR PHYSICIAN COMMUNICATION ABOUT TREATMENT

The patient behavior discussed in the section above represents not what patient-centered care advocates described as a goal when they worked to shift the tide of health policy but what nonetheless arose from that tide shift. In this section we discuss some of the consequences of higher patient involvement for physicians’ communication practices, with a focus on three areas. First, there is greater emphasis on treatment than on diagnoses today, likely in response to patient preoccupations with solutions, either at the expense of diagnosis or in addition to other issues a given patient has come in for. Second, treatment recommendations now involve more varied formats including those that overtly solicit patient involvement (e.g., proposals such as “How would you feel about trying *X?*” or suggestions like “Why don’t you start *Y*?”). Third, today’s clinicians orient to having greater accountability to patients with less authority to simply make pronouncements. This is done, for instance, through explaining to patients how they arrived at their diagnoses or why they are ordering a particular test.

### The Treatment Imperative

While treatment has long been prominent in health care, patients’ desires for solutions to problems means that as patient participation has increased, so has pressure for solutions: the treatment imperative (Shim et al. 2008). Kaufman (2015, p. 226) posited that the treatment imperative is driven by changes to the US health care system in which technoscientific innovations have created a demand for treatment “by any means, at any cost.” Consequently, diagnosing may be becoming less significant to patients and doctors alike.

In classifying the “diagnostic moment” as a relevant social action, Jutel (2014) set a foundation for subsequent work by contrasting the patient’s sensemaking of their own diagnosis based on perceptions of the doctor’s behavior with the specific action of diagnosis delivery from the doctor (e.g., “You have *X*”). While the diagnostic moment may be critical in certain types of care—such as oncology, where whether or not patients have cancer is very much what they want to know—in primary care visits, there may be a higher premium on problem solving.

In primary care, diagnoses and their uptake have been found to be surprisingly infrequent; this finding suggests that many doctors may be prepared to move on to the next activity without these elements (Heritage & McArthur 2019). Similarly, patients do not hold doctors accountable for providing diagnoses. In a sample of medical visits analyzed by Heritage & McArthur (2019), diagnoses were delivered in just over half (53%) of acute care visits. Of these, 30% were delivered without physician gaze at the patient, and 66% involved hedges in their delivery (e.g., “maybe,” “could be”). Subsequent uptake from patients most commonly involved only minimal response (59% of cases).

According to these data, in the slot typically designed for communicating diagnoses, doctors moved to discuss treatment instead. This suggests that treatment discussions take priority over diagnostic discussions. Thus, in these relatively mundane visits, there is an observed shift in medical practice toward treatments that address symptom burden, which stands in contrast to earlier work that affirmed the importance of diagnosis (Byrne & Long 1976, Robinson 2003). Heritage & McArthur (2019) proposed that a greater orientation to treatment over diagnosis in these visits may reflect physician orientation to patient signaling. This further suggests a shift toward greater patient involvement in the visit is partly responsible for these changes in physician behavior.

Other research also supports the idea that treatment is prioritized in a variety of medical visits. White (2020) observed the emergence of what she termed “noticings” in outpatient surgical consultations. Noticings are news announcements by doctors of new, unexpected problems unrelated to the primary condition for which patients are seeing their doctors. White found that noticings of additional concerns were formulated as delicate diagnostic news, and she concluded that while noticings can be beneficial for health outcomes insofar as they can support early detection and treatment of future problems, reassure patients, and strengthen the doctor-patient relationship, providing unsought news of trouble is not necessarily welcome. As this work and that of Heritage & McArthur (2019) suggest, the delivery of diagnoses may be both less formal and emerge in unexpected places to encourage treatment of other medical problems. In both instances, doctors orient themselves to addressing and treating patient problems over the diagnostic moment.

Not only is there a greater focus on treatment during clinical encounters, but the way in which physicians present treatments to patients also reflects a growing orientation toward the need to persuade patients to accept recommendations, sometimes by using unexpected strategies. For instance, Tate (2020) identified doctors positively invoking death in the clinical encounter (“You might die from this cancer”) as a bargaining chip when patients were resistant to treatment. This was done in the context of both early-stage and advanced-stage cancers; in the latter context, the recommended treatments were not necessarily curative. Conversely, though, Gill (2019) found that physicians invoked death as unlikely (e.g., “breast cancer won’t kill ya in the breast”) among early-stage cancer patients as an interactional on-ramp, which similarly underscores the importance of agreeing to treatment for the cancer and anticipates misconceptions patients may have at the time of a cancer diagnosis.

Even in the context of terminal disease, where more treatment is not necessarily beneficial to patients’ quality of life, there is support for the claim that treatment is still an imperative. Maynard and colleagues (2016) examined conversations between providers and end-stage lung cancer patient–caregiver dyads collected as part of an e-health intervention. They found that after delivering scan results showing patients evidence of their terminal disease, doctors provided positive inventories of treatments pursued thus far, which appeared primarily to facilitate transition to further treatment talk with patients (Singh et al. 2017). Using the same data set, Cortez et al. (2019) observed physicians’ reliance on comments about patient treatment not as a resource for dwelling on prognoses but rather as a device that actually bypasses prognostic discussions. They concluded that these statements can even lead into further treatment talk rather than allowing patients to demonstrate receipt and understanding of their terminal prognosis. Thus, today’s focus on treatment in medical care appears to be at least partly driven by patients playing a greater role in treatment decision making.

### Physicians’ Reliance on Participatory Treatment Formats

Identifying appropriate treatment for a medical problem and getting patients on board are critical components of treatment adherence and improving health outcomes (Mechta Nielsen et al. 2018, Schoenthaler et al. 2018). In data from the 1960s, physicians appeared primarily to rely on authoritative pronouncements about treatment, such as “Take this to the chemist” or “You need to take this medicine.” While pronouncements remain common in primary care (Stivers et al. 2018), treatment recommendations nowadays reflect a diverse array of actions and formats that nearly always work to provide more opportunities for patient participation through giving options, framing the treatment as a suggestion, and/or explicitly soliciting patient feedback. While this is what shared-decision-making advocates indicated as desirable, there are unintended consequences of these turn designs as well.

In some clinical contexts, such as neurology, clinicians may offer treatment recommendations as options—one way of explicitly orienting to the role patients should play in deciding treatment (Chappell et al. 2018, Toerien et al. 2011). However, while option listing may appear to provide patients choices, it can also constrain patient choice when doctors indicate their own preferences, rule out options, or make a case for or against an option. In oncology, Tate & Rimel (2020) found that doctors approach option listing differently depending on the patient’s stage in treatment (new versus existing problem) and whether standard options remain available. They concluded that when presenting treatment options to new patients with standard options remaining, as with neurology, doctors commonly reveal one option as more desirable and either implicitly or explicitly recommend one option over others. By contrast, options are listed without physician preference when patients’ existing treatments have been found ineffective. This means that most often, several unweighted options tacitly signal a poor prognosis to patients.

Relatedly, singular treatment recommendations are now more varied in terms of formats that involve patients as well. Stivers et al. (2018) established a typology of five treatment recommendation actions drawn from US and UK primary care visits, arguing that each action orients to differing levels of patient involvement and physician authority in decision making about treatment. While, as mentioned above, pronouncements (e.g., “I am starting you on *X*”) are most common, proposals (e.g., “Let’s try *X* and see how it goes”), suggestions (e.g., “You could try *X*”) that originate with the physician but frame the decision as shared, offers (e.g., “If you’d like I can give you *X*”), and even assertions (e.g., “*X* can work well”) are present as well.

Collaborative treatment recommendation action formats (proposals, suggestions, assertions, and offers) are more commonly associated with treatment resistance. For instance, proposals and offers, typically framed as collaborative between doctor and patient, yield more patient resistance in psychiatric consultations than do suggestions, possibly because the greater hedging in proposals and offers allows for patients to weigh in (Thompson & McCabe 2018). It is also possible that doctors use more collaborative recommendation actions in contexts where resistance is anticipated from prior knowledge of the patient.

Tate (2018) found that in oncology visits, doctors orient to patient agency as an important component of recommendations for initial treatment decisions and decisions about ancillary problems, like nausea or dizziness, and rely heavily on participatory recommendation formats. However, patient involvement is treated as less central to follow-up care decisions. In this environment, doctors rely primarily on pronouncements. This may suggest that physicians are more likely to rely on the participatory treatment formats when they anticipate resistance rather than the participatory formats engendering resistance. However, this remains a possible interpretation, as underscored by Opel et al.’s (2013) finding that pediatricians who lead into recommendations for vaccinations with pronouncements (e.g., “He needs three shots today”) versus more participatory statements (e.g., “Are you comfortable with doing three shots today?”) are more likely to secure parent agreement to vaccinations.

Thus, participatory treatment recommendations are increasingly common. However, they are consistently associated with greater resistance, though it remains unclear whether that is due to engendering resistance or to physician usage in contexts where resistance is more likely. The formats of treatment recommendations are only beginning to be investigated in other countries, but it is interesting to note that in Brazil, Ostermann found that participatory formats are quite lacking (Ostermann 2021), while in Britain, they are quite common (Stivers & Barnes 2018).

### Increased Physician Accountability

Providing patients with greater insight into clinical reasoning constitutes a third signal that physician behavior has been affected by the shift toward patient-centered care. While providing a diagnosis may seem to involve maximal physician authority (e.g., “You have *X*”), when physicians couple their diagnoses with accounts for why they believe their diagnosis is correct, they reduce their authority in the sense that they provide patients insight into their thinking (Peräkylä 1998). Physician accounts offer patients greater opportunity to question diagnoses, particularly if a patient is expecting one kind of diagnosis and receives another. While previous studies predominantly concluded that doctors delivered diagnoses in authoritarian ways, Perakyla (1998) offered evidence of a shift toward more patient-centered forms of communication because, in doing so, physicians open up their reasoning and hence their authority.

Standardized testing for diagnosis also reflects increasing physician accountability to patients. In these cases too, research shows that physicians increasingly offer patients and families insight into the reasons behind diagnoses. Maynard & Turowetz (2017) argued that physicians work to produce “accountable” test results for autism spectrum diagnoses, sometimes changing test questions or procedures in subtle ways. Specifically, children are evaluated for their degree of “abstract competence” when posed hypothetical questions. Testers work to ensure that a test result showing low levels of abstract competence is not merely an artifact of the test by reasking questions. In this way, testers orient to the need to be able to defend their test results for a given child in subsequent discussions. Yet, in doing so, they also shape test outcomes.

In this section, we have discussed three key ways that the context of high patient involvement in health care has shaped physicians’ treatment-relevant communication: a greater focus on treatment, heavier reliance on participatory treatment recommendation designs, and increased physician orientation to accountability. These behaviors are often positive changes. For instance, participatory formats for treatment recommendations are associated with increased patient participation. However, that participation is most likely to be resistance. As patient participation increases, physicians need tools for talking with these engaged patients to avoid compromises in care quality when the patients lobby for outcomes that are at odds with best care practices.

## COMMUNICATION TOOLS FOR WORKING WITH ENGAGED PATIENTS

The paradigms of shared decision making and patient-centered care idealize collaborative physician–patient partnerships where patients voice their preferences and values in decision making while doctors are obliged to consider available medical evidence about which treatment to pursue. In the sections above, we have discussed some ways that engaged patients are changing treatment outcomes. In this section we review what is known about three communication domains: securing and maintaining patient engagement, managing pressure for inappropriate diagnoses and treatments, and entering into discussions of difficult diagnoses and prognoses.

### Managing Patients’ Concerns

While patients are participating more than ever, they can still leave with unaddressed concerns. Research has shown that the ways that physicians question patients can support the detection and eventual diagnosis of medical problems that may be important but not the reason for seeking care. Patients bring, on average, three concerns to a primary care visit, but a typical agenda-setting question at the start of a visit (e.g., “What can I do for you today?”) will often solicit just one concern (Heritage et al. 2007). In an interventional study, two different question types were analyzed: the positively tilted “Is there something else you’d like to discuss? ” and the negatively tilted question “Is there anything else you’d like to discuss?” Positively polarized questions increased patients’ likelihood of raising additional issues and reduced unmet needs compared with negatively polarized questions.

Along similar lines, Robinson et al. (2016) used a secondary data set of 407 acute primary care visits to explore the relationship between types of agenda-setting solicitations and patient communication of additional concerns. The authors examined the position of the solicitation (early or late in the visit), the solicitation type (concern seeking versus question seeking), and whether patients responded with additional concerns. They found that agenda-setting questions of any sort were done relatively infrequently: in only 18% of visits. Concern-seeking questions (e.g., “Do you have other concerns/issues?”) produced early in the visit were the most likely to solicit additional patient concerns, and concern-seeking questions were more likely to solicit additional concerns in contrast to solicitations of questions (e.g., “Do you have other questions?”).

### Steering Patients Toward Acceptance

There are some medical conditions for which multiple treatment options have similar risks and similar success rates. In these cases, patient preferences are usually critical for the decision. However, for many encounters, there is a clear standard of care for a patient’s condition, and patient lobbying for an alternative, or resistance to the recommendation, may represent a request for inappropriate or inferior care. In these cases, communication resources that increase rates of patient acceptance are critical to any hope of subsequent patient adherence to recommendations.

As discussed above, patients sometimes resist diagnoses or treatments for acute conditions. Physicians can preempt resistance by offering a running commentary of their findings—particularly unproblematic findings. When clinicians use this “online commentary” during visits in which they are disinclined to offer antibiotics for upper respiratory tract symptoms, parents are less likely to resist the nonantibiotic treatment recommendation (Heritage & Stivers 1999, Mangione-Smith et al. 2003).

Another resource for securing acceptance is the language that clinicians use in talking about patients’ problems. When patients present mental health problems in primary care, they are more likely to resist a physician’s treatment recommendation if the physician has used different language to describe the problem than the patient used earlier in the visit (McCabe 2021). For instance, a patient who accounted for her problem as stress was subsequently faced with a physician recommendation for an antidepressant. The patient resisted this recommendation and treated the clinician’s understanding of her condition as not shared (i.e., the clinician recommended treating depression rather than anxiety) regardless of what the clinician’s rationale might be. Patient acceptance in self-harm presentations also appears to be shaped by a highly patient-attentive, tailored discussion of the problem (Bergen & McCabe 2021).

Physicians can also facilitate diagnostic acceptance through their method of mobilizing clinical reasoning. While we have discussed above how physicians are increasingly orienting to their own accountability when explaining diagnoses to patients (Perakyla 1998), Turowetz & Maynard (2016) took this one step further to show how fitting patients into diagnostic categories can support diagnostic acceptance through a method they termed “category attribution.” Category attribution, developed in the context of diagnosing a child with autism, is a discursive process in which a narrative about a set of abstract behaviors—fitted to clinical categories—is used to support the clinical rationale for an autism diagnosis. Turowetz and Maynard argued that such a process supports diagnostic acceptance and treatment adherence.

Research has also found that preempting patient resistance to the treatment can be accomplished through the format of the treatment recommendation. Ruling out a treatment (e.g., “This isn’t something an antibiotic would cure”) offers no solution to a patient’s problem, whereas an affirmative recommendation (e.g., “So you need to give Robitussin DM several times per day”)—particularly a recommendation that provides a concrete next action step—does. In the context of upper respiratory tract infections, affirmatively designed treatment recommendations are less likely to be resisted than those that are solely designed negatively (Mangione-Smith et al. 2006, Mangione-Smith et al. 2015, Stivers 2005a). This concept likely transfers to other types of encounters where patients will respond better to being given something that they can do for their problem rather than to being told what they cannot do.

### Delicate News Delivery

A third area where health communication researchers have made inroads in advantageous strategies related to treatment outcomes involves high-stakes clinical contexts where patients may have a severe or life-threatening condition and may be facing a change in prognosis. In these cases, the communication tools doctors use are distinct from those for steering patients toward acceptance. This is partly because in many cases there is a less clear treatment path forward. Patients often disbelieve and deny bad news, or hear bad news but maintain a positive outlook by focusing on uncertainty, but eventually they can come to understand the terminality of their disease (Glaser & Strauss 1965, Timmermans 1994). Thus, in these contexts, the communication goal is acceptance of the situation (and often a lack of curative treatment) rather than of a particular treatment or lifestyle change.

Lutfey & Maynard (1998) found that in discussing a terminal patient’s prognosis, the use of perspective-display sequences can be an effective way to ease patients into acceptance of their prognoses. In these cases, rather than clinicians telling patients what the prognosis is, clinicians solicit the patient’s opinion about their current health status and help them to articulate the bad news themselves before offering their own clinical views (Maynard 1989).

In UK palliative care, Pino et al. (2016) observed a related practice, “elaboration solicitation,” which ushers in the topic of end-of-life (EOL) care by drawing on patients’ earlier talk rather than introducing it as a new topic. The authors found that elaboration solicitations take the form of fishing questions (“What’s going on in your head when your pain is bad? ”) or “You said”–prefaced paraphrases in which providers ask patients to say more about information they offered earlier in the visit. This practice helps providers avoid the perception that they are pushing EOL care options on patients and instead facilitates patient involvement in accepting the terminality of their illness. Using the same palliative care data set, Land et al. (2019) found that doctors use “hypothetical scenario sequences” to redirect patients’ expectations toward a more truthful, and possibly upsetting, future outcome. These sequences were found to supplement patients’ positions about treatment rather than negate them while also gently redirecting patients’ expectations toward the eventual progression of their illness.

Pino & Parry (2019) relatedly focused on patient-initiated practices in prognostic news sequences. Their analysis revealed that UK hospice patients seek life-expectancy estimates from their doctors through statements rather than overt questions (e.g., “How long do I have?”). The authors proposed that these elaborated statements reflect a patient’s willingness to learn more, anticipate that doctors may not be able to provide an exact estimate, and summarize what the patient may already know about their illness trajectory. Doctors, too, were found to support an environment that prepares patients for difficult news before delivering it, such as asking patients about their prior knowledge and whether they are ready to hear such an estimate.

Delivering difficult prognostic news delicately supports patient engagement, trust, and adherence. While some may argue that being direct and honest with patients about prognosis is ethical, clinicians also call for gently delivering bad news over time rather than up front because this facilitates patient trust, which leads to improved communication about care (Helft 2005). A trusting relationship between doctor and patient is critical in making decisions aligned with patients’ goals, values, and wishes (Csikai & Martin 2010). Greater trust in doctors can facilitate delicate decisions with patients, such as referrals to palliative care (Hay et al. 2017) and hospice (Cicolello & Anandarajah 2019). Evidence also suggests that trusting doctor–patient relationships lead to reduced patient resistance in delicate contexts; for instance, it has been found that more trusting relationships with providers support patients’/caregivers’ acceptance of and adherence to hospice care as treatment (Russell et al. 2020, Waldrop & Meeker 2012).

Overall, when doctors initiate delicate news delivery to highly engaged patients, they run the risk of upsetting their patients and disengaging them from their care, thus compromising the doctor–patient relationship and goals of treatment. The work in this section demonstrates that the interactional tools doctors use to deliver such news are distinct insofar as they attempt to maximize patient involvement by being responsive to patients’ observations in second position. From there, doctors can coax patients to recognize delicate news on their own.

## DISCUSSION

The physician–patient relationship has seen a tremendous change in the past 100 years from a time when medical authority reached extreme highs, and patients were relatively passive, to a time when patients are highly engaged and clinician–patient relationships are increasingly egalitarian (Timmermans 2020). In this article, we have argued that while this change in the nature of medical encounters has a variety of positive consequences, some consequences are problematic and risk pitting quality of treatment outcomes against patient participation.

We have discussed patient engagement as not constrained to indicating preferences between two types of treatments that are similar in outcomes and risks. Rather, patients are also advocating for treatment, including medication that is not clinically indicated, through the ways in which they present their problems, respond to history-taking questions, add information, and take up diagnoses and treatment recommendations. Through all of these means, research has documented that patients can alter the course of treatment outcomes.

Moreover, further changes in the physician–patient relationship and communication are visible through the consequences of patient engagement on physician behavior. Physicians increasingly place more emphasis on treatment than diagnosis, rely on more participatory formats for delivering recommendations, and are more oriented to their own accountability than in decades past. While these behaviors are generally positive, the jury is still out on whether these same behaviors are actually encouraging patients to resist physician advice and are thus increasing physician–patient conflict over treatment decisions.

We have concluded with a review of research about how to reduce conflict between patients and physicians in the era of engaged patients. Specifically, as we have discussed, much can be done early in the consultation to minimize opportunities for conflict, including solidifying an agenda that includes all patients’ concerns and foreshadowing diagnoses, treatments, and prognoses when clinicians can anticipate that they may be met with resistance, distress, or dissatisfaction.

We believe that to improve physician–patient communication about treatment and maintain or improve quality of care, physicians need more communication strategies for working with engaged patients. It is also worth considering the impact that institutional structures designed to engage patients have on outcomes. As part of its “Triple Aim,” the Institute for Healthcare Improvement identifies both care quality and patient satisfaction as central to the patient experience of care (Berwick et al. 2008). As such, improving patient experience ratings is important to clinicians. Many health care systems link patient satisfaction on surveys with provider financial incentives (Mehta 2015). In the case of opioid prescribing, providers may face a dilemma: satisfy patients with pain control that may not be warranted, or recommend a nonopioid treatment? Physicians who receive incentives for higher patient satisfaction report an impact on their prescribing patterns three times more often than those who do not have incentives (Carrico et al. 2018). Provider-rating sites in which patients may comment on their experiences with their doctor in a public forum, like Zocdoc or Healthgrades, may further compound the need for providers to satisfy their patients to maintain their practice at the expense of care quality. While there may be countermeasures in place to monitor overprescription of opioids, no such measures exist for inappropriately prescribing other drugs or for recommending procedures outside the standard of care. This adds further complexity to the changing dynamic of involving patients in decision making.

This conflict represents a chief risk in the shift to a more egalitarian physician–patient relationship. At times, conflict can be a productive way for physicians to learn about patient priorities, preferences, and concerns and can lead to physician recommendations that are attentive to patient needs and more likely to be followed. However, much of the conflict that occurs in clinic visits concerns tensions regarding patient preferences against taking medicine that has clear evidence of benefit (e.g., high cholesterol medication or antiseizure medication) or in favor of taking medications that have no clear evidence of benefit (e.g., taking an antibiotic for a protracted cold).

In this review, we have focused primarily on US health care. In part, this is because the vast majority of research based on video recordings of physician–patient interactions is in this system. Where we could, we have included the select research on physician–patient interaction in other countries (e.g., Brazil, China, Japan, and the United Kingdom) that examines patient resistance and negotiation. Ostermann’s (2021) work in Brazil documented a system where physicians still have much more medical authority than in the United States. Wang and colleagues’ work in China (e.g., Wang & Liu 2021) suggested that physicians have much less medical authority. Thus, it is important that future work explore other health care systems.

## CONCLUSION

We are at a critical juncture in health communication. At present, medical authority in the United States is still largely preserved. Although the dynamic is far more egalitarian than it was between the 1950s and 1970s, there are still many indicators that patients are not (yet) prepared to see their physicians as simply providers of a service. For instance, although patients are engaged in many communication practices to advocate for treatments or diagnoses, they still rarely do this overtly and show much hesitancy to outright request or demand medications, referrals, tests, etc. Similarly, when they do tread into clinicians’ domains, they commonly do so with delicate language, orienting to their own reticence to do so.

However, if patients are increasingly tipping the scales toward desired treatments and other outcomes in ways that run counter to best health care practices, we may soon see a negative effect on life expectancy outcomes. Because we want to maintain patient involvement in care, we must address the problems this raises in other ways. Thus, scholars of health communication should examine this area both for structural changes that reduce dilemmas for physicians and for further communication strategies that can be used to manage patient pressure during visits—particularly in settings not yet explored, such as specialty care, inpatient settings, and urgent care settings.
